# Urinary Nephrin Levels Among Pregnant Women With Preeclampsia in Lagos, Southwest Nigeria: An Analytical Cross-Sectional Study

**DOI:** 10.7759/cureus.49472

**Published:** 2023-11-27

**Authors:** Ayodeji A Oluwole, Tolulope T Fasesin, Adeyemi Okunowo, Gbenga Olorunfemi, Kehinde S Okunade

**Affiliations:** 1 Obstetrics and Gynaecology, College of Medicine, University of Lagos, Lagos, NGA; 2 Obstetrics and Gynaecology, Lagos University Teaching Hospital, Lagos, NGA; 3 Epidemiology and Biostatistics, School of Public Health, University of the Witwatersrand, Johannesburg, ZAF

**Keywords:** lagos, urinary nephrin, preeclampsia, diagnostic marker, lmic

## Abstract

Background: Hypertensive disorders in pregnancy are one of the leading causes of maternal and perinatal morbidity and mortality worldwide. The clinical utility of urinary nephrin as a diagnostic biomarker of preeclampsia is currently of research interest. However, this is yet to gain significant traction within clinical settings.

Objectives: We evaluated the association between maternal urinary nephrin levels and the occurrence and severity of preeclampsia among pregnant women in Lagos, Nigeria.

Design: We conducted an analytical cross-sectional study involving pregnant women diagnosed with preeclampsia as well as their age- and gestational-age-matched normotensive counterparts. We tested the association between high maternal urinary nephrin levels and the occurrence of preeclampsia without and with severe features. P < 0.05 was reported as statistically significant.

Results: The study showed that for every unit increase in urinary nephrin levels, the odds of preeclampsia increased by about ninefold (adjusted Odds ratio = 8.9, 95% confidence interval: 2.8-29.2, P < 0.001). The levels of urinary nephrin increased steadily with increasing severity of the disease: 1.9 ± 0.8 ng/mL in preeclampsia without severe features, 2.7 ± 0.7 ng/mL in preeclampsia with at least one severe feature, and 3.3 ±1.1 ng/mL in eclampsia.

Conclusion: There was an association between elevated levels of urinary nephrin and preeclampsia and its severe variant. However, there is a need for more robust studies with a longitudinal characterization of urinary nephrin levels to establish causal relationships with preeclampsia, explore other potential risk factors of preeclampsia, and define the clinical usefulness of urinary nephrin as a potentially reliable and accurate predictive marker of preeclampsia among women in low- and middle-income countries (LMIC) settings.

## Introduction

Hypertensive disorders of pregnancy constitute one of the leading causes of maternal and perinatal mortality worldwide [1] and remain a significant problem in sub-Saharan Africa, where the highest disease burden is reported [2,3]. It is reported to complicate about 2%-8% of all pregnancies globally, with varying incidences from place to place [4,5]. In Nigeria, the incidence varies between 4% and 16% [5,6]. Preeclampsia is a pregnancy-specific multisystemic disorder characterized by the occurrence of gestational hypertension and significant proteinuria after 20 weeks of gestation in a previously normotensive and non-proteinuric woman [1]. Although new-onset proteinuria is usually a prominent feature, preeclampsia may also be diagnosed in some women in the absence of proteinuria [5,7]. In this context, such women will present with hypertension and any of the following severe features: new-onset headache unresponsive to acetaminophen and not accounted for by alternative diagnoses or visual disturbances, pulmonary edema, severe persistent right upper quadrant or epigastric pain that is not accounted for by alternative diagnoses, thrombocytopenia (platelet count less than 100 x 10^9^/L), impaired liver function as indicated by abnormally elevated blood concentrations of liver enzymes (to twice the upper limit of the normal concentration), and renal insufficiency (serum creatinine concentration greater than 1.1 mg/dL or a doubling of the serum creatinine concentration in the absence of other renal diseases) [1].

Clinicians have always classified pregnant women during antenatal risk assessments based on their historical risk of developing preeclampsia using risk factors such as preexisting medical disorders, family history, increased maternal age, and parity [6]. However, most women with risk factors usually do not develop the disease. It is, therefore, pertinent to identify an ideal biomarker for accurate early detection of preeclampsia as this will offer a window of opportunity to introduce interventions for reducing disease progression and complications [3]. However, despite many decades of extensive research, the pathophysiology of preeclampsia remains unclear [8]. This is because of the heterogeneity of the disorder and its varying pathogenesis from woman to woman [9]. Various mechanisms have been implicated in the development of preeclampsia, and these include placental hypoxia/ischemia, immunological maladaptation between maternal decidua and fetal trophoblasts, and altered maternal inflammatory response [8]. Another important mechanism is the imbalance between pro- and anti-angiogenic factors such as vascular endothelial growth factor (VEGF), placental growth factor (PIGF), and soluble fms-like tyrosine kinase-1 (sFlt-1), which results in maternal endothelial dysfunction [9].

The production of urinary nephrin is important in the pathogenesis of proteinuric preeclampsia, and several studies have examined its clinical usefulness as a marker of renal dysfunction [8-10]. Podocyte injuries, as well as tubular dysfunction, occur in preeclampsia with severe feature(s), and therefore, reduced shedding of nephrin after delivery is an indicator of improved renal function in a woman who had preeclampsia [10]. Urinary nephrin excretion is recognized as a marker of early glomerular injury, and there is now increasing interest in its measurements as a marker of disease prediction and improved renal functional recovery [11,12]. Nephrinuria precedes albuminuria and appears to have a positive correlation with the severity of preeclampsia [11,12]. Therefore, the use of urinary nephrin as an early diagnostic marker of preeclampsia may have advantages over clinical assessments based on risk factors, blood pressure, and urinary protein measurements, which are usually evidence of established severe disease.

Studies have examined the role of urinary nephrin in preeclampsia, but findings from these studies are yet to be applied to mainstream clinical practice, especially in the low- and middle-income countries (LMICs) with the highest burden of the disease [9,12,13]. No study, to the best of our knowledge, has been conducted in the sub-Saharan African setting. This current study, therefore, was aimed at evaluating the clinical applicability of a cheap and non-invasive biomarker in a resource-limited setting by evaluating the association between urinary nephrin and the occurrence of preeclampsia among women in Lagos, Nigeria.

## Materials and methods

Study design and setting

This analytical cross-sectional study was carried out among preeclamptic pregnant women and their normotensive counterparts at the accident and emergency unit, labor ward, and antenatal clinic of a University Teaching Hospital in Lagos, Nigeria, between September 2020 and July 2021. The hospital is affiliated with a College of Medicine and is the largest government-owned tertiary institution in Lagos. It serves as the referral center for most private and state-owned hospitals in Southwest Nigeria. The obstetric unit of the hospital conducts an average of 170 deliveries per month and manages an average of 10-20 cases of preeclampsia each month.

Study population and case definitions

Eligible participants for the case group were consecutively consenting pregnant women diagnosed with preeclampsia at 26-40 completed weeks of gestation, and their comparison group comprised consecutively selected age- and gestational age-matched normotensive women. Women with multiple pregnancies and those with a known medical condition such as hypertension, renal disease, diabetes mellitus, systemic lupus erythematosus, HIV, or other infections were excluded from participation. Preeclampsia is defined as new-onset hypertension characterized by systolic blood pressure ≥ 140 mmHg or a diastolic blood pressure ≥ 90 mmHg measured using an appropriate-sized cuff on two different occasions taken at least four to six hours apart with or without proteinuria (≥300 mg in 24 hours urine or ≥2+ on dipstick urinalysis done on at least two random urine samples taken at least four to six hours apart) after the first half of pregnancy [1,14]. Severe preeclampsia is defined as systolic blood pressure ≥ 160 mmHg and/or diastolic blood pressure ≥ 110mmHg with proteinuria ≥3+ on dipstick urinalysis or preeclampsia with any evidence of clinical and/or laboratory dysfunction [1,14]. Evidence of clinical dysfunction includes headache, photophobia, and epigastric/hypochondria pain, while laboratory dysfunctions are polycythemia, low platelet count, deranged renal function with elevated uric acid, hemolysis, and elevated liver enzymes and low platelets (HELLP) syndrome [14,15]. The study endpoints are the associations between high levels of urinary nephrin and the occurrence and severity of preeclampsia.

Sample size determination

Using the data from a previously published study by Son et al. [16] with a standard deviation (σ) of 0.23 multiple of median (MoM), a unit normal deviate that corresponds to the desired type 1 error rate at a 95% confidence interval (Zα/2) of 1.96, a desired type II error rate (Zβ) of 5% (1.64), and between-group mean difference in urinary nephrin levels of 4.1 MoM, we estimated that a minimum sample size of 32 women would be required in each study group based on a non-response rate of 20%. Therefore, a total of 64 participants comprising 32 preeclamptic and an equal number of healthy normotensive women as comparators were enrolled in the study.

Participants' recruitment and data collection

Eligible women were enrolled consecutively into the study after obtaining informed written consent upon counseling and explaining the purpose and procedure of the study. We used a pretested interviewer-administered structured questionnaire, data on the participant's sociodemographic status, gestational age at enrolment, and body mass index (BMI - calculated as the actual pre-gestational or first-trimester maternal weight in kilograms divided by the square of height in meters). We based gestational age on the first day of the participant's last menstrual period and/or first or early second-trimester dating ultrasound scan. About 10 mL of midstream or catheter urine samples were obtained from each participant and then collected in universal bottles before being transferred to the hospital’s main laboratory within one hour for analysis. A serial number was assigned to each specimen bottle to conceal the identity of the participants from the laboratory scientists. The urine samples were centrifuged at 2,000-3,000 revolutions per minute for 20 minutes, after which the supernatant was stored at -20°C until assayed for nephrin concentration by the chemical pathologist, who was blinded to the participant's clinical status. Urinary nephrin was analyzed using the solid-phase sandwich human nephrin-enzyme-linked immunosorbent assay (NPHN-ELISA) kit (Bioassay Technology Laboratory, China).

Statistical analyses

Data analyses were performed using Stata version 16 (StataCorp., College Station, TX) statistical software. Descriptive statistics were computed for relevant sociodemographic and clinical data, and continuous variables were tested for normality using the Kolmogorov-Smirnov test with Lilliefors' significance correction. Associations between categorical variables were compared using Pearson's χ^2^ test, while continuous variables were tested using the independent sample t-test for normally distributed data or the Mann-Whitney U-test for skewed data. Analysis of variance (ANOVA) test was used to compare the levels of urinary nephrin in normotensive women and across the spectrum of preeclampsia. The association between maternal urinary nephrin levels and preeclampsia was tested using univariable logistic regression analysis, following which variables with a P-value < 0.20 were built into a multivariable model. Statistical significance was reported at P < 0.05.

## Results

As shown in Table 1, there were statistically significant differences in parity (P = 0.025), booking status (P = 0.001), systolic (P = 0.001), and diastolic BP (P = 0.001) between the preeclamptic and their normotensive comparison groups. There were no differences in the participants’ age (P = 0.821), BMI (P = 0.092), and ethnic group (P = 0.344).

**Table 1:** Sociodemographic and clinical characteristics of study participants (n = 64) GA: Gestational age.

Characteristics	Preeclamptic	Normotensive	P-value
	n = 32	n = 32	
Mean GA at enrolment (weeks)	32.5 ± 5.1	34.6 ± 4.8	0.092
Age (years)	30.7 ± 6.0	30.4 ± 4.9	0.821
Parity	1.0 (0.0-2.0)	3.0 (0.0-2.0)	0.025
Median BMI (kg/m^2^)	30.5 (21.7-31.6)	28.8 (22.6-32.5)	0.092
Systolic blood pressure (mmHg)	165.3 ± 20.2	120.6 ± 12.3	0.001
Diastolic blood pressure (mmHg)	104.5 ± 15.5	73.6 ± 8.3	0.001
Ethnicity			0.344
Yoruba	17 (53.1)	15 (46.9)	
Igbo	8 (25.0)	13 (40.6)	
Others	7 (21.9)	4 (12.5)	
Booking status			0.001
Booked	11 (34.4)	27 (84.4)	
Unbooked	21 (65.6)	5 (15.6)	

**Figure 1:**
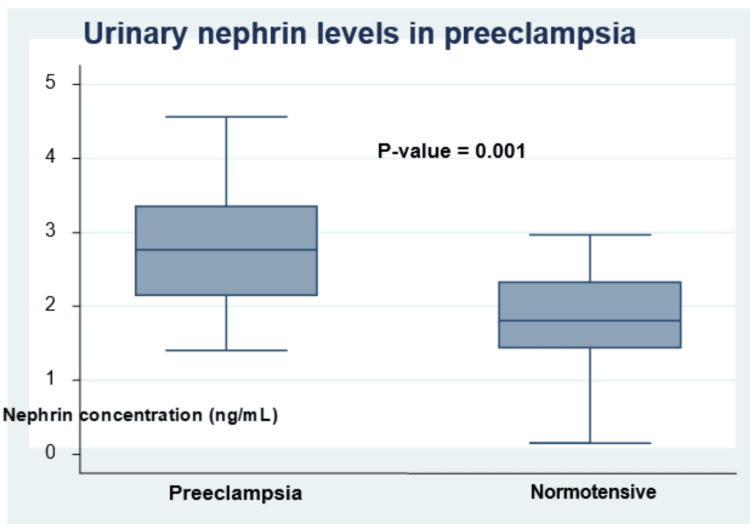
Box plot showing the urinary levels of nephrin in preeclamptic and normotensive pregnant women (2.76 ± 0.75 vs 1.73 ± 0.74 ng/mL, P = 0.001)

The mean urinary nephrin level was higher among the preeclamptic participants than in their normotensive counterparts (2.8 ± 0.8 vs 1.7 ± 0.7 ng/mL, P = 0.001) as shown in Figure 1.

After a multivariate analysis to control for possible confounders such as age, parity, and BMI (Table 2), for every unit increase in urinary nephrin levels, the odds of preeclampsia increased by about ninefold in the study participants (adjusted Odds ratio = 8.9, 95% CI: 2.8-29.2, P < 0.001).

**Table 2:** Univariable and multivariable logistic regression of the association between urinary nephrin levels and preeclampsia CI: Confidence interval; OR: Odds ratio.

Factors	Univariate			Multivariate		
	Crude OR	95% CI	P-value	Adjusted OR	95% CI	P-value
Nephrin levels in ng/mL	7.6	2.6–21.9	0.001	8.9	2.8–29.2	0.001
Age in years	1.0	0.9–1.1	0.818	1.1	0.9–1.2	0.399
Body mass index						
Normal	1.0	Reference	-	1.00	Reference	-
Overweight	2.9	0.5–18.4	0.253	1.8	0.2–21.6	0.647
Obese	1.5	0.2–9.5	0.688	1.3	0.1–15.1	0.833
Parity						
Nulliparity	1.0	Reference	-	1.0	Reference	-
Multiparity	0.5	0.2–1.4	0.211	0.4	0.1–1.6	0.190

**Figure 2:**
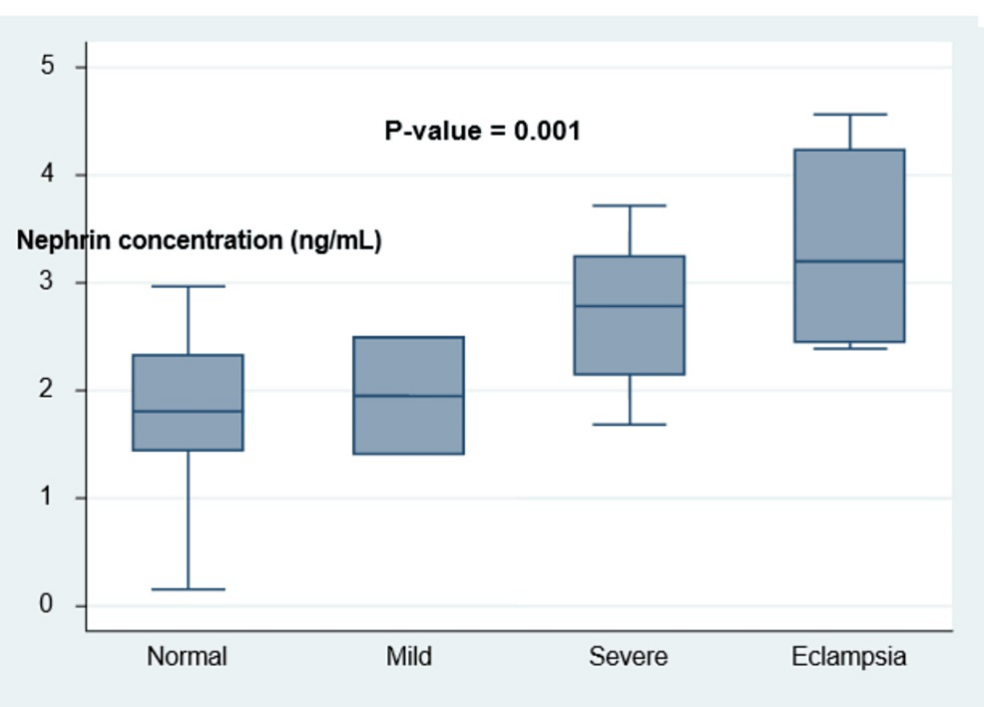
Box plot showing the urinary levels of nephrin from normotensive (1.7 ± 0.7 ng/mL) to all spectrum of preeclampsia from mild (1.9 ± 0.8 ng/mL) and severe preeclampsia (2.7 ± 0.7 ng/mL) to eclampsia (3.3 ± 1.1 ng/mL) (P = 0.001)

The levels of urinary nephrin increased steadily with disease severity from 1.9 ± 0.8 ng/mL in women with preeclampsia without severe features, 2.7 ± 0.7 ng/mL in women diagnosed as having preeclampsia with at least one severe feature, and 3.3 ± 1.1 ng/mL in women who had eclampsia (Figure 2).

## Discussion

This study assessed and compared the urinary nephrin levels in women with preeclampsia and their normotensive counterparts in the third trimester of pregnancy and found a ninefold increase in the urinary nephrin concentration in women with preeclampsia compared to their normotensive counterparts. We also reported a steady increase in the urinary concentrations of nephrin with increasing severity of preeclampsia.

The mean age of 30 years reported in our study reflects the trend in the urban Lagos setting, where women usually pursue their professional advancements while delaying reproduction until around the third decade of life [17]. This is similar to the mean age of 31.9 ± 4.5 years [3] and 30.3 ± 5.2 years [17], which is reported in similar cohorts of women enrolled in the same clinical setting in Lagos. Nulliparity has been previously identified as an important risk factor for preeclampsia due to the lack of immunological competence between fetoplacental and maternal tissues [18], and our finding, in similarity to another Lagos study [6], corroborated this theory by observing a significant association between nulliparity and preeclampsia. Our study showed a significant association between a woman’s booking status and preeclampsia. This is a common phenomenon in most LMIC settings where the lack of antenatal care does not allow for a woman’s risk identification and classification before the institution of preventive intervention in early pregnancy.

We reported a significant association between nephrinuria and preeclampsia with(out) severe feature(s), suggesting that an increased urinary nephrin shedding, which reflects damage to the glomerular slit diaphragm, contributes to the development of proteinuria, a good indicator of renal damage [6,11,16]. In our study, urine nephrin concentration was also associated with the severity of preeclampsia, supporting the logical explanation that disease severity is characterized by high diastolic and systolic blood pressure as well as significant proteinuria indicative of kidney damage, and will also be similarly associated with increased nephrin excretion. This is further demonstrated in our study, where urinary nephrin levels increased with increasing severity of preeclampsia, which is comparable to the findings from other studies worldwide [10,11,16].

Our study has some limitations. First, the sample size was relatively small and, therefore, was not powered to detect the difference in urinary nephrin concentration in preeclamptic women with or without severe features, thus limiting the generalizability of the findings. Second, the study was cross-sectional in design, which means that causal relationships cannot be established. Third, the study did not explore preexisting medical conditions as potential risk factors for preeclampsia in our analysis. Fourth, the study did not assess the potential impact of treatment on urinary nephrin levels. Finally, the study was conducted in a single center, which may limit the generalizability of the findings to other settings. However, we have been able to add to the body of knowledge and have generated hypotheses for further testing in future longitudinal studies that will assess the performance accuracy of urinary nephrin as a predictive biomarker of preeclampsia and its severity in a cohort of Nigerian women enrolled in the first trimester of pregnancy.

## Conclusions

This study showed higher levels of urinary nephrin in preeclamptic women than in normotensive pregnant women, and there was no association between elevated levels of urinary nephrin and disease severity. Thus, this suggests a possible future role for urinary nephrin as a potential screening and diagnostic marker of preeclampsia in LMICs. However, there is a need for more robust studies with a longitudinal characterization of urinary nephrin levels to establish causal relationships with preeclampsia, explore other potential risk factors of preeclampsia and define the clinical usefulness of urinary nephrin as a potentially reliable and accurate predictive marker of preeclampsia among women in LMIC settings.
